# Supplementary material for: Identification of a Novel Gene MtbZIP60 as a Negative Regulator of Leaf Senescence through Transcriptome Analysis in Medicago truncatula
Source: Int J Mol Sci. 2024 Sep 27;25(19):10410. doi: 10.3390/ijms251910410 (PMC11477300; doi:10.3390/ijms251910410)
Supplement: Supplementary file 1 [file ijms-25-10410-s001.zip › Supplemental Table S1-Revised.pdf]

Table S1. Primers used in this study

Primers	Sequences 5’-3’	Annealing temperature	cycle number	Application
MtbZIP60-*Xba*I-F	AGAGAACACGGGGGACTCTAGAATGGCTTCATCAAGTGGAACAT	58°C	35	To amplify DNA of *MtbZIP60* for construction of overexpression vector
MtbZIP60-*BamH* I-R	AGATTAGCTTTTGTTCGGATCCGTACTGCAGTATATCTGCAGAAG			
MtbZIP60-BD-*Nde*I-F	TCAGAGGAGGACCTGCATATGGCTTCATCAAGTGGAACAT	58°C	35	To amplify DNA of *MtbZIP60* for yeast two-hybrid
MtbZIP60-BD-*BamH* I-R	CCGCTGCAGGTCGACGGATCCTCAGTACTGCAGTATATCTGCAG			
LTR6	GCTACCAACCAAACCAAGTCAA	58°C	35	Primers in *Tnt1* for genotyping of different mutant
LTR31	CTCCTCTCGGGGTCGTGGTT			
MtWRKY40-BD-*EcoR* I-F	GGAATTCATGGATTTTTCATCATCATGG	58°C	35	To amplify DNA of *MtWRKY40* for yeast two-hybrid
MtWRKY40-BD-*BamH* I-R	CGGGATCCCTAATTCTGGTGTAACATTTT			
MtbZIP60-qF	GCAAGGCGGTCTAGGATGAG	60°C	45	For quantitative RT-PCR analysis of *MtbZIP60*
MtbZIP60-qR	ATTCTCCCATCTGAGCCCTC			
MtbZIP60-NF4038-F	CGCAGGACCCATTTAGTTCA	58°C	35	For genotyping of NF*4038*
MtbZIP6-NF4038-R	ATCCGTCTCAGTACTGCAGT			
MtbZIP60-*Kpn* I-nLucF	CGGGGTACCATGGCTTCATCAAGTGGAACAT	58°C	35	To amplify DNA of *MtbZIP60* for LCI assays
MtbZIP60-*Sal* I-nLucR	ACGCGTCGACGTACTGCAGTATATCTGCAGAAG			
MtWRKY40-*Kpn* I-cLucF	CGGGGTACCATGGATTTTTCATCATCATGG	58°C	35	To amplify DNA of *MtWRKY40* for LCI assays
MtWRKY40-	ACGCGTCGACCTAATT			

Sal I-cLucR	CTGGTGTAACATTTT			
MtWRKY40-qF	ATGGATTTTTCATCATCATGG	60°C	45	For quantitative RT-PCR analysis of *MtWRKY40*
MtWRKY40-qR	GCCTTGGTGGAGGAGCTGAAA			
MtbZIP60-AD-*Nde*I-F	GGGAATTCCATATGGCTTCATCAAGTGGAACAT	58°C	35	To amplify DNA of *MtbZIP60* for yeast two-hybrid
MtbZIP60-AD-*BamH* I-R	CGGGATCCGTACTGCAGTATATCTGCAGAAG			
MtCAB1-qF	CAACCACCAAGAAGAACGTA	60°C	45	For quantitative RT-PCR analysis of *MtCAB1*
MtCAB1-qR	TGACCTCGAGTTCACGGTTC			
MtORE1-qF	ATGAGGTTGGATTCACTTGGT	60°C	45	For quantitative RT-PCR analysis of *MtORE1*
MtORE1-qR	AGTTGGAGAAGCAGGACACG			
